# Microstructure Evolution and Mechanical Behavior of 2219 Aluminum Alloys Additively Fabricated by the Cold Metal Transfer Process

**DOI:** 10.3390/ma11050812

**Published:** 2018-05-16

**Authors:** Xuewei Fang, Lijuan Zhang, Hui Li, Chaolong Li, Ke Huang, Bingheng Lu

**Affiliations:** 1 School of Mechanical Engineering, Xi’an Jiaotong Univeristy, No. 28, Xianning West Road, Xi’an 710049, China; fangxuewei0801@163.com (X.F.); ke.huang@xjtu.edu.cn (K.H.); bhlu@mail.xjtu.edu.cn (B.L.); 2 National Innovation Institute of Additive Manufacturing, Building A, Door of Metropolis, Jinye Road, Gaoxin District, Xi’an 710065, China; lihui@niiam.cn (H.L.); lichaolong@niiam.cn (C.L.)

**Keywords:** cold metal transfer, 2219-Aluminum, additive manufacturing, porosity, microstructure, mechanical property

## Abstract

In this research, four different welding arc modes including conventional cold metal transfer (CMT), CMT-Pulse (CMT-P), CMT-Advanced (CMT-ADV), and CMT pulse advanced (CMT-PADV) were used to deposit 2219-Al wire. The effects of different arc modes on porosity, pore size distribution, microstructure evolution, and mechanical properties were thoroughly investigated. The statistical analysis of the porosity and its size distribution indicated that the CMT-PADV process gave the smallest pore area percentage and pore aspect ratio, and had almost no larger pores. The results from optical microscopy, scanning electron microscopy, and fractographic morphology proved that uniform and fine equiaxed grains, evenly distributed Al_2_Cu second phase particles were formed during the CMT-PADV process. Furthermore, the X-ray diffraction test ascertained that the CMT-PADV sample had the smallest lattice parameter and the highest solute Cu content. Besides, the tensile strength could reach 283 MPa, the data scattering was the smallest, and the strength scattering of the sample in the horizontal direction was the shortest. In addition, the strength properties were nearly isotropic, with only 5 MPa difference in the vertical and horizontal directions. The above mentioned results indicated that the mechanical properties of 2219 aluminum alloy was improved using the CMT-PADV arc mode.

## 1. Introduction

In the past two decades, additive manufacturing (AM) has become more and more popular in the manufacturing field for direct fabrication of metal components. AM technologies for metals can be classified into powder-fed and wire-fed based on the form of feedstock unit [1,2,3,4]. Among the metal AM methods, wire and arc additive manufacturing (WAAM) techniques have now expanded the metal AM market for fabricating customized and large-scale metal components with high deposition rate and a relatively low building and investment cost [5]. WAAM is a process that combines electric arc as the heat source and wire as the feedstock. It was investigated for the AM purpose from the 1990s. WAAM has been recognized as one of the most efficient AM methods, which allows fabrication of large components up to several meters, such as cruciforms, stiffened panels, and wing ribs [6]. A number of different arc welding processes, such as gas metal arc welding (GMAW), gas tungsten arc welding (GTAW), and plasma arc welding (PAW), have been applied to fabricate metal parts [7,8,9].

GMAW is a well-known welding and AM process that generates an electric arc between a consumable wire electrode and the substrate. During deposition, the wire is normally perpendicular to the forming substrate. GTAW and PAW use a non-consumable tungsten electrode to produce the metal parts. The wire-fed orientation plays a key factor in obtaining a steady and smooth welding bead [10]. By contrast, GMAW has advantages of high productivity and convenience for automation. Compared with powder-based AM, WAAM has a lot of advantages, for instance, high material efficiency, high deposition rate, no stringent requirement for gas shielding, low capital cost, and absence of powder-related troubles [11]. To improve the heat input and energy density of the GMAW process, a modified MIG welding process named the cold metal transfer (CMT) process was invented by the Fronius company [12]. The CMT process is a relatively new method which is characterized by its low heat input and high deposition rate. What’s more, CMT is a spatter-free droplet transfer process conducted via precise control of current parameters and wire movement during the short-circuit transfer. A specially designed welding torch holding a very powerful motor can reach a high frequency forward and backward movement (up to 130 Hz) of the wire during welding, which can reduce the energy input to a minimum degree [13].

The basic theory of CMT has been studied by making a comparison between CMT and the conventional MIG process [14]. A heat source model to simulate the effect of periodic and recurrent arcing and metal deposition phenomena in the cold metal transfer type of welding was established [15]. It was reported that CMT is suitable for welding aluminum alloy sheets due to its low heat input, high deposition rate, and small deformation [16]. Over the years, aluminum structural parts have been widely used in aerospace, high-speed rail trains, and the automobile manufacturing industry. Especially, high strength aluminum alloys have become more and more important in the aerospace and automobile industries. To meet modern industry requirements of size and shape complexity for metallic components, researchers have focused on the metal AM process recently and WAAM has been proved as an economic alternative method for fabricating aluminum components. The effect of the arc mode in the CMT process on the porosity characteristics of Al–Cu alloy has been investigated [17]. The mechanical properties of 2219-Al components produced by additive manufacturing with TIG method was studied by Bai et al. [18]. Al-4043 and Al-5356 alloys have been produced by variable polarity gas tungsten arc welding [19,20]. 

However, different materials show different characteristics during the welding process. Although the typical porosity analysis of the CMT process on welding Al–Cu alloys has been studied [17], the effects of the arc modes on the mechanical properties, microstructure evolution, and the pore characteristics on fabricating 2219 aluminum alloy remain unknown. In this paper, 2319-Al wire was chosen as the raw material to investigate the effect of different CMT arc modes on the porosity, microstructure, and mechanical properties of the AM process. Different arc modes and adjusting the wire feed speed were used to find out the optimal process parameters for the additive manufacturing of 2319-Al alloys. The effects of different arc modes on pore numbers, pore size distribution, pore aspect ratio, microstructure evolution, and mechanical properties were discussed in detail.

## 2. Experimental Procedure

**Figure 1:**
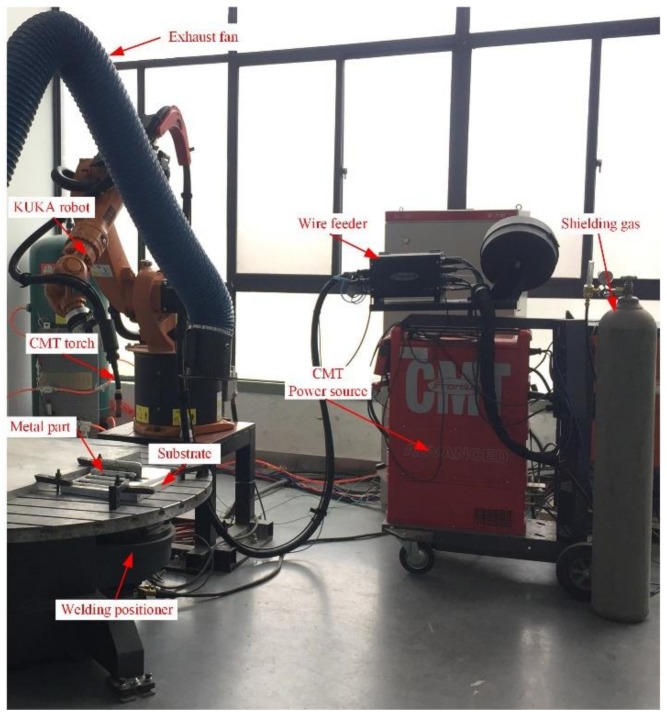
The CMT (Cold metal transfer)–based WAAM experimental platform for 2219 aluminum alloy.

**Figure 2:**
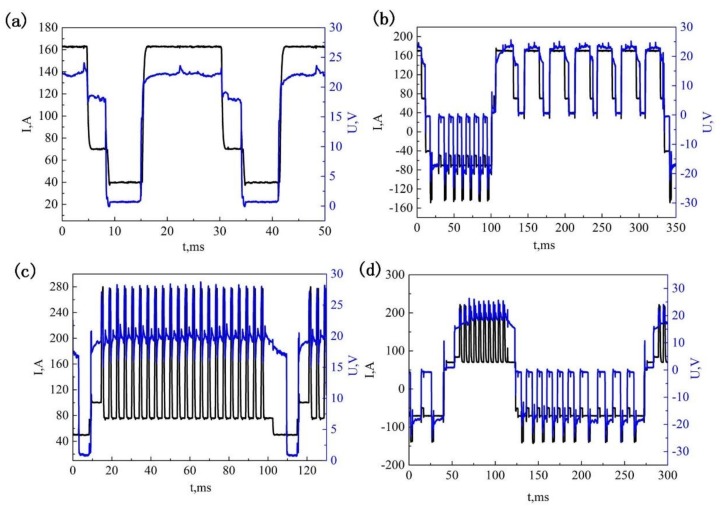
Measurements of arc current and voltage waveforms of (**a**) CMT; (**b**) CMT-ADV; (**c**) CMT-P, and (**d**) CMT-PADV.

The compositions of the commercial ER2319 (ESAB, West Lebanon, NH, USA) and 2219-T87 aluminum (CHALCO, Chongqing, China) plates used in the study are listed in Table 1. The substrates were washed in alkaline water and dried in air, followed by mechanical cleaning and degreasing using acetone immediately before deposition. A Fronius CMT Advanced 4000 R (Fronius, Pettenbach, Austria) and a KUKA KR16 robot (Kuka, Augsburg, Germany) were employed in the present study, as shown in Figure 1. Thin wall samples were prepared in four different arc modes, which are conventional CMT, CMT-ADV, CMT-P, and CMT-PADV, respectively. Pure argon (99.999%) was used as the shielding gas with a constant flow rate of 25 L/min. The contact tip to work distance was fixed at 15 mm. The wire feed speed (*WFS*) and travel speed (*TS*) of the four arc modes used for the sample building are given in Table 2, and the actual arc current and voltage waveforms measured for these four modes are shown in Figure 2.

**Figure 3:**
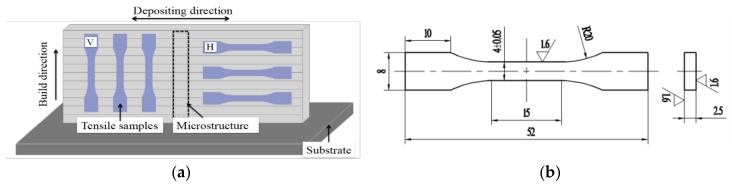
(**a**) Schematic graph of the sampling positions; (**b**) the dimensions (mm) of tensile sample.

Samples for testing were sectioned as shown in Figure 3a. Ten mm of both ends of each wall were cut off and discarded. The tensile test samples were machined as shown in Figure 3b. The tensile test was conducted following the ISO 6892-1-2009 standard. The tensile tests were carried out at ambient temperature in an electro-mechanical universal testing machine with a constant tensile speed of 1 mm min^−1^. Fracture surfaces morphology of tensile test specimens were observed by scanning electron microscopy (SEM, SU3500, Tokyo, Japan). Specimens for microstructure analysis were mechanically taken from the middle part of the wall samples, then polished to a mirror finish. Optical microscopy (OM) (DM2700M, LEICA, Wetzlar, Germany) and SEM were employed for taking images for the microstructure examination. Continuous frames of porosity images were analyzed by Image-Pro Plus (IPP, 5.1, Media Cybernetics, MD, USA) software. OM and SEM Specimens were etched with Kroll’s reagent. An X-ray diffractometer (D8 Advanced, BRUKER, Karlsruhe, Germany) was used for XRD phase analysis. The scanning angle ranged from 20° to 90°.

## 3. Results and Discussion

**Figure 4:**
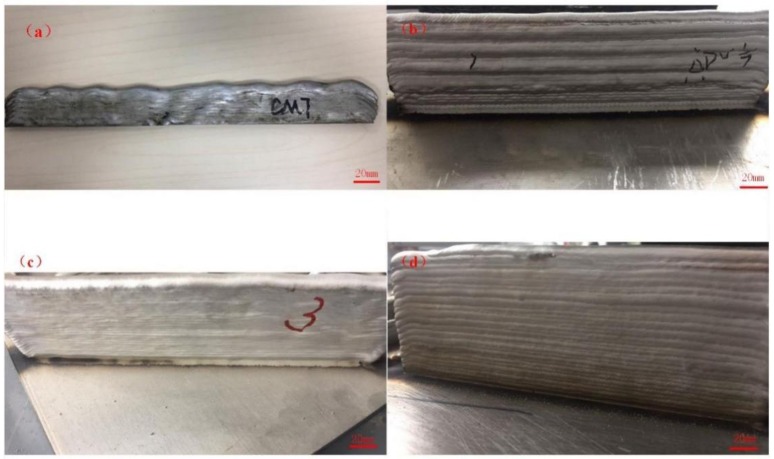
The multilayer additively manufactured 2219 aluminum wall samples using different arc modes: (**a**) CMT; (**b**) CMT-ADV; (**c**) CMT-P, and (**d**) CMT-PADV.

As shown in Figure 4, the multilayer additively manufactured 2219 aluminum wall samples were produced by different arc modes. It can be seen that the height of wall was all about 30 mm and the top surface of the sample using the CMT mode was wavy and uneven (Figure 4a). Cong et al. pointed out that conventional CMT is not suitable for the additive manufacturing process because it produces a large amount of gas pores, even in single layer deposit [17]. The wall geometry using CMT-ADV process was also irregular (Figure 4b). During the CMT-ADV process, the droplet transfer formation was not stable when fabricating the sample, which resulted in poor wall geometry. Figure 4c shows the multilayer additive wall produced by the CMT-P mode; the surface of the wall was smooth and uniform. A better wall profile was obtained by the CMT-PADV process, as shown in Figure 4d, and the average layer height was about 2.2 mm. Researchers have proposed various theories to explain why humping occurs and the fundamental mechanisms of formation were analyzed. Mendez et al. [21] proposed a theory of the arc induced humping based on the pressure and heating ranges of the arc. Based on the arc pressure model, good welding can be achieved by selecting reasonable parameters to reduce the arc pressure.

### 3.1. Microstructure

#### 3.1.1. Optical Micrographs

**Figure 5:**
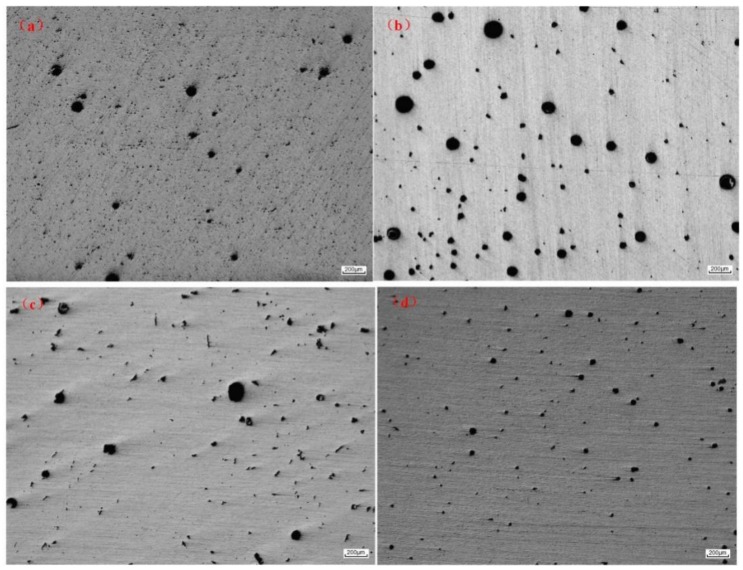
Optically observed porosity for the WAAM 2219 aluminum alloys by different modes: (**a**) CMT; (**b**) CMT-ADV; (**c**) CMT-P, and (**d**) CMT-PADV.

Figure 5 illustrates the distributions of gas pores in samples produced in four different modes. The pores distributed in the WAAM 2219 alloys were in a wide range. In order to better understand the relationship between the pore distribution and the arc modes, the total number, mean pore diameter, area percentage, and mean aspect ratio of the pores were calculated. The measurements were carried out from four fields of the sample and observed at a magnification of 50 times. The observation position was from the middle of the sample and four captures were taken continuously from the bottom to the top. The results are shown in Table 3. Mean pore diameter (*D*) is defined as the diameter of a circle with equivalent area, where *A_m_* is the measured cross-sectional area of the pore. The mathematical equation can be expressed as Equation (1):

(1) $$D=\sqrt{\frac{4A_{m}}{\pi }}$$

Besides, pore morphology was also investigated by image analyzer in terms of aspect ratio, defined as the ratio of maximum to minimum dimension of pores. It represents a perfect aspect ratio if the aspect ratio is equal to 1. However, a large amount of precipitates were distributed in the 2219 aluminum alloy, and the size of the precipitates was small. When the observed object was less than 5 μm, the optical microscopy used in the article cannot distinguish the pores and the precipitated phases. Only pores larger than 5 μm in size were counted.

As shown in Table 3, it can be seen that the arc mode had a significant influence on the porosity. Compared to the other three modes, the conventional CMT process produced the largest number of gas pores. The CMT-ADV process significantly reduced the number of gas pores but the size of pores was increased. In addition, the mean diameter of the pores produced by CMT-ADV process was the largest with the size of 30.0 μm and some pores were even larger than 100 μm in diameter. The CMT-P and CMT-PADV processes further reduced the number and size of the gas pores. For the CMT-PADV process, the pore area percentage was the smallest at only 0.98%. The average aspect ratio for the CMT and CMT-P was 3.76 and 4.69, respectively. In comparison, the aspect ratios of the CMT-ADV and CMT-PADV processes were significantly reduced, indicating that the pores had grown to be more spherical. It could be concluded that the CMT-PADV arc mode is the most suitable process for depositing aluminum alloys with a controllable porosity.

**Figure 6:**
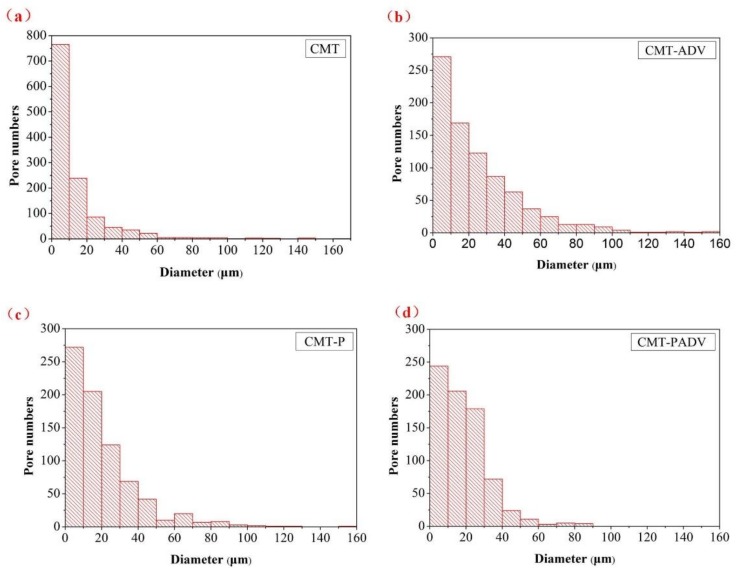
Pore size distribution in numbers for the 2219 aluminum alloys fabricated by different arc modes: (**a**) CMT; (**b**) CMT-ADV; (**c**) CMT-P, and (**d**) CMT-PADV.

**Figure 7:**
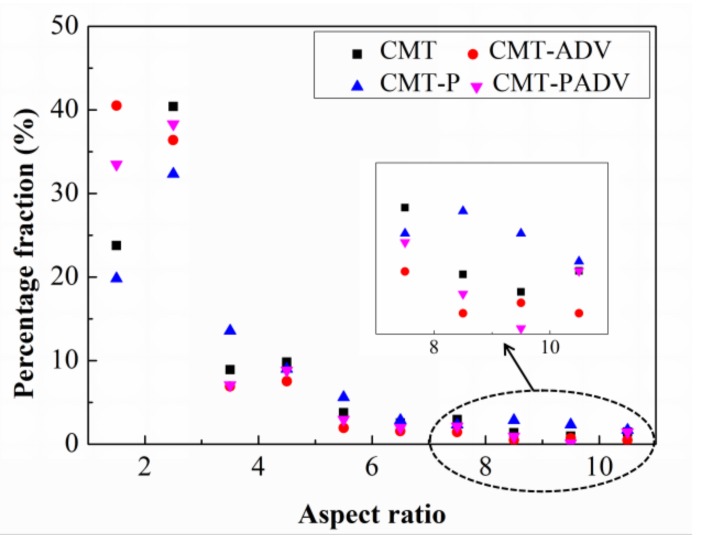
Pore aspect ratio distribution in percentage fraction for the 2219 aluminum alloys fabricated in different arc modes.

As shown in Figure 6 and Figure 7, the detailed comparisons were performed by sorting the pores with different diameters and aspect ratio. Figure 6 shows the pore size distribution by sorting the number of pores in diameter with an interval of 10 μm. In all samples, most of the gas pores were small pores (from 5 μm to 20 μm). Compared to other modes, the CMT process produced the largest number of small pores, and the number of pores exceeded 1200 in the observed area. In contrast, the number of these small pores was reduced by about 56.2%, 52.5%, and 55.2% for the CMT-ADV, CMT-P, and CMT-PADV processes, respectively. Large pores (more than 100 μm) were observed in samples produced by CMT, CMT-ADV, and CMT-P modes which has a detrimental influence on the mechanical properties. In particular, the pore size distribution in CMT-ADV was relatively wide (from 5 μm to 160 μm) which proved this arc mode was not stable for depositing 2219-Al. In comparison, there were absolutely no gas pores larger than 100 μm in 2219 alloy deposited by the CMT-PADV arc mode.

The percentage fractions of pores with small aspect ratio (from 1 to 2) for the CMT-ADV and CMT-PADV modes made up a large proportion (Figure 7). Also, it can be observed that the proportion of pores with large aspect ratio (from 7 to 11) for the CMT-ADV and CMT-PADV mode was very small, as shown in the enlarged area, which indicated that the pores tend to be more spherical in these two arc modes. The above results demonstrated that the CMT-PADV was suitable for the deposition of 2219 alloys.

**Figure 8:**
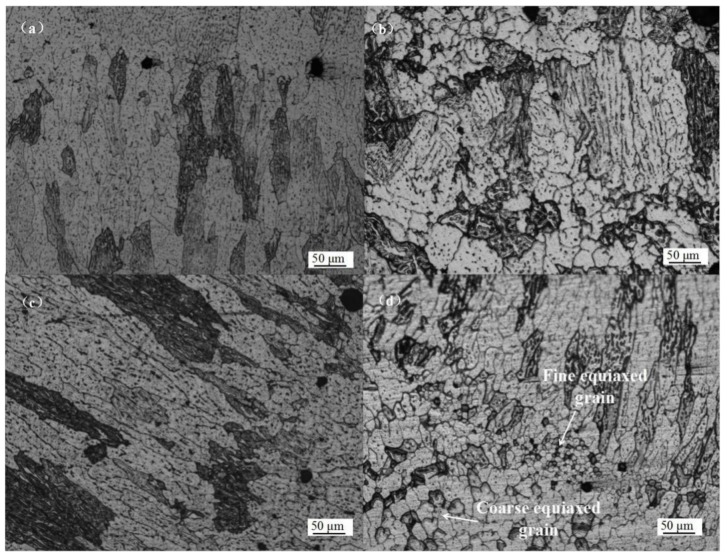
Microstructure of the WAAM 2219 alloys using different modes: (**a**) CMT; (**b**) CMT-ADV; (**c**) CMT-P, and (**d**) CMT-PADV.

Figure 8 illustrates the microstructure of longitudinal sections of a multilayer wall built in the four different arc modes. During the CMT and CMT-P process, a coarse columnar grain structure was observed, as shown in Figure 8a,c, respectively. There was a distinct fusion zone between layers. For the CMT-ADV process, it can be observed that equiaxed grains were distributed in the fusion zone (Figure 8b). Compared with the other three modes, a mixture of coarse columnar grain and finer equiaxed grain structure was observed while using CMT-PADV as shown in Figure 8d, the latter consisted of fine and coarse equiaxed grains. 

It is known that the grain size and morphology are affected by the heat input (*HI*). The heat input for different trials were calculated using Equation (2):

(2) $$HI=\eta \;\times \left(\sum U_{i}I_{i}\right)/TS$$

where *U_i_* and *I_i_* are the arc voltage and current for the building of the 2219-Al samples. *η* is the arc thermal efficiency of CMT and is set to 0.8 here. As can be seen from Table 3, the PADV mode had the least amount of heat input among the four arc modes. The suitable *HI* can effectively refine the grain structure and promotes the formation of equiaxed grains. It is speculated that some phase particles existing in the filler wire, such as Al_3_Ti and Al_3_Zr, can be preserved during the CMT-PADV process due to its lower *HI*. Guo et al. found that intermetallic aluminides of Ti and Zr could provide heterogeneous nucleation sites for α(Al), resulting in a microstructure with uniform and fine equiaxed grains [19].

#### 3.1.2. SEM Results

**Figure 9:**
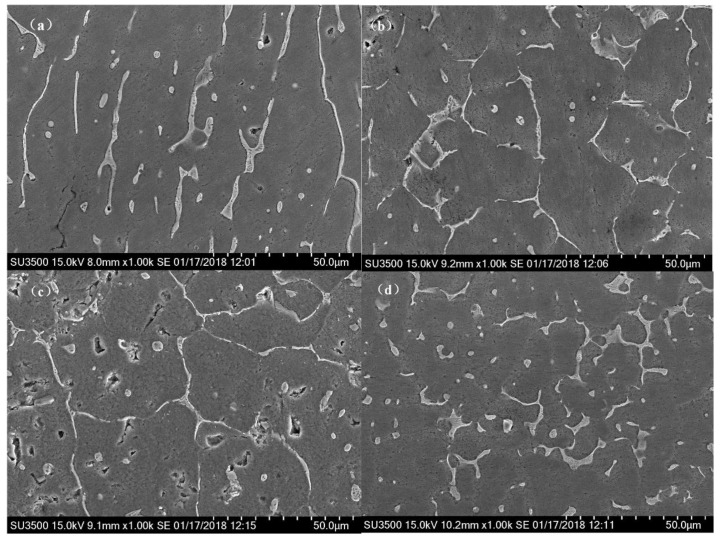
Scanning electron micrographs for the 2219 aluminum alloys using different arc modes: (**a**) CMT; (**b**) CMT-ADV; (**c**) CMT-P, and (**d**) CMT-PADV.

SEM images of the WAAM 2219 alloys using different arc modes are shown in Figure 9. It can be seen that the white network-like second phase particles scattered along the grain boundaries and distributed in the transgranular regions, as shown in Figure 9a–c. These second phase particles can easily coarsen and were unevenly distributed due to the decreased cooling rate during the repeated thermal cycles of the WAAM process. Besides, this segregation generally causes Cu solute atoms forming a depletion zone around the phase, which will negatively impact the mechanical properties. Compared with other three modes, the second phase particles were more evenly distributed in the matrix during the CMT-PADV process (Figure 9d).

#### 3.1.3. XRD Results

**Figure 10:**
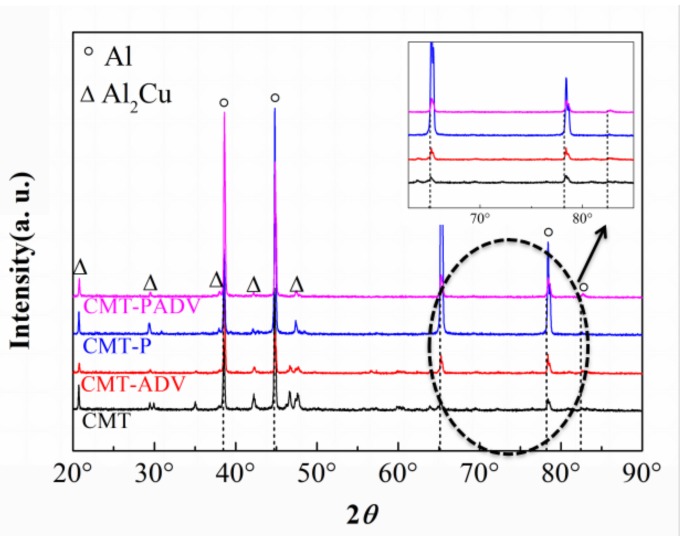
XRD patterns of the WAAM 2219Al alloys using different arc modes.

The XRD patterns of 2219 alloys deposited in different arc modes are shown in Figure 10. Characteristic peaks representing α-Al and Al_2_Cu phases can be indexed in all samples, which agreed with traditional 2219 alloy fabricated by welding.

In Figure 10, the theoretical diffraction peaks of pure Al are indicated by vertical dotted lines. The refined measurement was done by Jade6 software to evaluate the lattice constants. It shows that, for the CMT, CMT-ADV, CMT-P, and CMT-PADV samples, the lattice constants were 4.0435 ± 0.0002, 4.0398 ± 0.0001, 4.0413 ± 0.0002, and 4.0373 ± 0.0006 Å, respectively. Obviously, these values were all smaller than the lattice constant for the pure Al phase (4.0494 Å).

The residual stress in the WAAM process was tensile stress in the non-equilibrium solidification process reported by Sun et al. The lattice constant would become larger under the tensile stress. But, all XRD peak positions actually shift to the high scattering angles which indicated that the lattice constant of the Al matrix becomes smaller according to the Bragg equation [22,23]. Therefore, the lattice reduction was mainly caused by the incorporation of Cu solute atoms, which have a smaller radius than Al, into the Al matrix.

Besides, the CMT-PADV mode had the least amount of heat input among the four modes, and the smallest heat input led to a higher solidification process which contributed to the suppression of Cu precipitation from the matrix. Therefore, there were fewer Cu atoms separated out to produce the continuous secondary precipitates along the grain boundaries which was beneficial in terms of the mechanical properties.

### 3.2. Tensile Properties 

**Figure 11:**
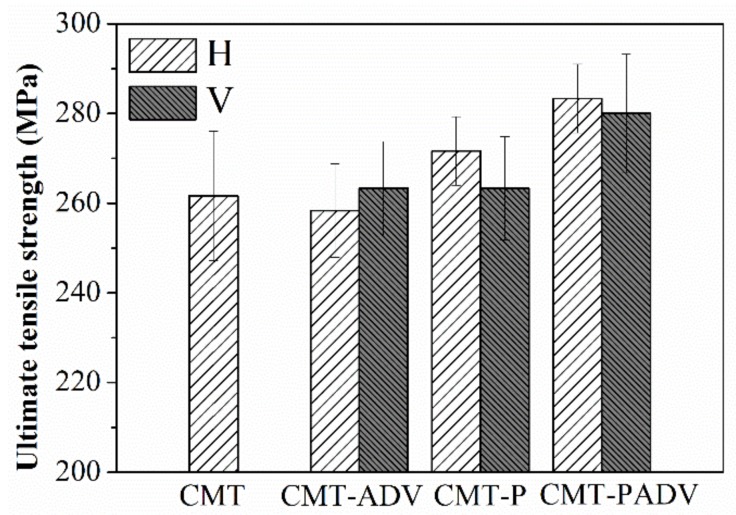
Tensile properties for the 2219 alloys fabricated by different arc modes.

The histograms in Figure 11 shown the ultimate tensile strength of samples using different arc modes, where H represents the horizontal direction and V represents the vertical direction. As mentioned above, the height of the wall was limited due to the poor formability of the conventional CMT process. Therefore, only the tensile strength in the horizontal direction was obtained and listed, which was 264 MPa. It was found that the tensile strength of the CMT-ADV process was slightly lower than that of the CMT process in the horizontal direction. During the CMT-P process, the tensile strength in the horizontal direction was increased to 270 MPa. Compared with the other three modes, the best tensile strength was obtained by the CMT-PADV mode, which could reached 283 MPa in the horizontal direction. Furthermore, the error bar of the data in the horizontal direction was the shortest, which indicated that the mechanical properties of 2219 aluminum alloys was improved by using the CMT-PADV arc mode. What’s more, the tensile properties were nearly isotropic with only a difference of 5 MPa in the vertical direction and horizontal direction. However, it can be found that the strength of the samples obtained by the CMT-P and CMT-PADV regimes in the horizontal direction was slightly higher than the analogous characteristics of samples cut in the vertical direction. As reported by Zhang et al., the anisotropy percentage of Al-6Mg alloy deposited by WAAM ranges from 8% to 27% [24]. The results showed that the pores and their size in the horizontal fractures were less than that of the vertical fractures. They believed that more pores lead to the instability of the vertical performance.

From the above analysis, it can be concluded that the CMT-PADV arc mode was the most suitable process for building 2219 aluminum alloys. Compared with other arc modes, the number and size of pores were significantly reduced by using the CMT-PADV arc mode. To a large extent, the reduction of pores may promote the excellent performance of mechanical properties. Donlon et al. pointed out that pores could act as preferential crack initiation sites [20], and Mayer et al. found that porosity would easily act as the initiation site of a crack if their mean diameters were greater than approximately 50–100 μm [25]. As reported by Kobayashi et al. (2010), pores could generate negative effects on the strength of aluminum [26]. This was because the porous regions probably yield first due to the reduced tensile load bearing capacity, causing stress concentration near the voids and then premature fracture may occur. In addition, the grain structure was also refined due to the low heat input of the CMT-PADV arc welding process. Furthermore, the XRD results show that the highest solubility of Cu atoms was obtained by using the CMT-PADV process, which could greatly improve the mechanical properties through solution strengthening. Besides, some phase particles which exist in the filler wire, such as Al_3_Ti and Al_3_Zr, can be preserved due to the low heat input of CMT-PADV process. Radović et al. reported that dispersoids of transition metal particles could effectively pin the grain boundaries to improve the mechanical properties [27].

**Figure 12:**
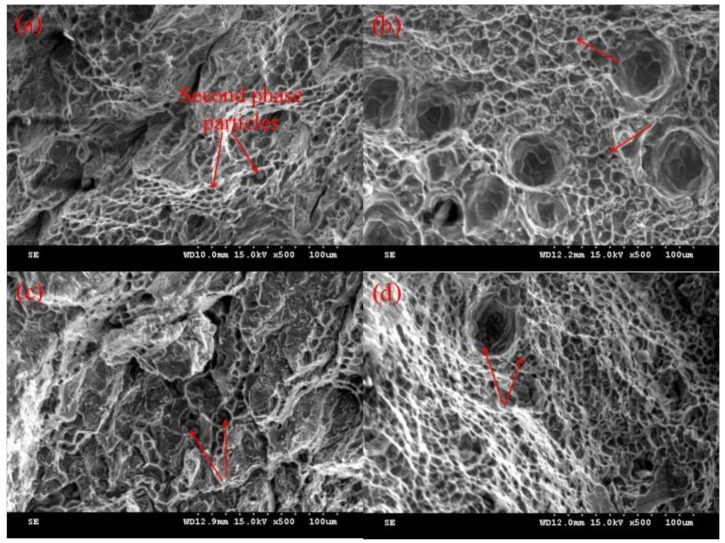
SEM fractograph morphology for tensile specimens of the WAAM 2219 alloys by different arc modes (**a**) CMT; (**b**) CMT-ADV; (**c**) CMT-P, and (**d**) CMT-PADV.

Figure 12 illustrates the SEM images of the tensile test fractograph morphology in the horizontal direction for the 2219 alloys built in different arc modes. From Figure 12a–d, all the fractured surfaces consist of dimples, which were considered an indication of ductile fracture. It can be seen that some second phase particles (indicated by arrows) were embedded at the center of the dimples, which can initiate primary cracks [28]. The Cu solute atom depletion zones (SDZ) in the as-deposited alloy were formed around grain boundaries due to the concentrated scattered Al_2_Cu particles along the grain boundaries [29]. These SDZ areas were weaker in strength compared to other areas. Under static tensile stress, the tearing edges surrounding the particles were generated at these SDZ areas. As shown in Figure 9, the second phase particles were more evenly distributed in the matrix during the CMT-PADV process, which contributed to improving the mechanical properties of 2219 aluminum alloys.

## 4. Conclusions

The effect of four arc modes on the porosity, microstructure, and tensile properties of cold metal transfer additively manufactured 2219-Al has been systematically investigated in this study. Results from the experiments and different kinds of tests can be used to draw the following conclusions.

1. The results showed that the 2219-Al wall fabricated CMT-P and CMT-ADV arc modes can generate smooth and uniform multilayer thin-wall parts.
2. The conventional CMT process produced the largest number of small gas pores. The mean diameter of the pores produced by the CMT-ADV process was the largest with the average size of 30.0 μm and some pores were even larger than 100 μm. However, In the CMT-PADV arc mode, the pore area percentage was the smallest (only 0.98%) and there were no gas pores larger than 100 μm. Equiaxed grains were distributed in the fusion zone and a mixture of coarse columnar grain and finer equiaxed grain structure was observed. In addition, the CMT-PADV samples have the smallest lattice parameter which indicates the highest solute level of Cu in Al alloys.
3. The best tensile strength was obtained by the CMT-PADV mode. The tensile strength could reach 283 MPa in the horizontal direction. What’s more, the strength properties were nearly isotropic with only a difference of 5 MPa in the vertical and horizontal directions. It can be concluded that will with proper control and monitoring of the process parameters in the CMT-PADV arc mode, large size 2219 Al parts with excellent properties can be rapidly deposited.

## Figures and Tables

**Table 1:** Nominal composition of ER2319 wire and the 2219-T87 substrate.

Alloys	Chemical Composition (wt %)								
	Cu	Mn	Mg	Ti	Zr	V	Zn	Si	Fe
ER2319	5.8–6.8	0.2–0.4	<0.02	0.1–0.2	0.1–0.25	0.05–0.15	<0.1	<0.2	<0.3
2219-T87	5.8–6.8	0.2–0.4	<0.02	0.02–0.1	0.1–0.25	0.05–0.15	<0.1	<0.2	<0.3

**Table 2:** Parameters for 2219-Al wall fabricated by four arc modes.

Arc Mode	Welding Parameters
Cold metal transfer (CMT)	*WFS* = 6 m/min *TS* = 0.5 m/min *HI* = (220.9 J/mm)
CMT-Advanced (CMT-ADV)	*WFS* = 6 m/min *TS* = 0.5 m/min *HI* = (194.0 J/mm)
CMT-Pulse (CMT-P)	*WFS* = 5 m/min *TS* = 0.5 m/min *HI* = (231.8 J/mm)
CMT pulse advanced (CMT-PADV)	*WFS* = 7 m/min *TS* = 0.5 m/min *HI* = (130.1 J/mm)

**Table 3:** Analysis results of pores for 2219 alloys fabricated in different arc modes.

Arc Mode	Number of Pores (In a Total Area of 34 mm^2^)	Mean Diameter (μm)	Area Percentage (%)	Mean Aspect Ratio
CMT	1220	14.4	1.33	3.76
CMT-ADV	822	30.0	2.60	2.94
CMT-P	767	21.4	1.52	4.69
CMT-PADV	747	18.9	0.98	3.24
